# Combined Effect of Neutron Radiation and Curcumin on Breast Cancer Cells Cytotoxicity in 3D Culture Medium

**DOI:** 10.52547/ibj.3756

**Published:** 2022-10-28

**Authors:** Sajedeh Zargan, Mehdi Salehi Barough, Jamil Zargan, Mohsen Shayesteh, Ashkan Haji Noor Mohammadi, Mohsen Mousavi, Hani Keshavarz Alikhani

**Affiliations:** 1 Department of Medical Radiation Engineering, Central Tehran Branch, Islamic Azad University, Tehran, Iran;; 2 Medical Radiation Research Center, Central Tehran Branch, Islamic Azad University, Tehran, Iran;; 3 Department of Biology, Faculty of Basic Sciences, Imam Hossein Comprehensive University, Tehran, Iran;; 4 Department of Nuclear Physics, Imam Hossein Comprehensive University, Tehran, Iran;; 5 Faculty of Science, Department of Biology, Razi University, Kermanshah, Iran

**Keywords:** Breast neoplasms, Curcumin, MCF-7 cells

## Abstract

**Introduction:**

Chemotherapy, biotherapy, and radiotherapy play a limited but important role in treating breast cancer. For more efficient treatment, combination therapy could be an appropriate option. In this study, radiotherapy using neutron radiation emitted from a ^241^Am-Be neutron source, as well as biotherapy using curcumin (80 μM) was combined to investigate the efficiency of treatment towards MCF-7 breast cancer in a 3D culture medium.

**Methods:**

MTT, NR uptake assay, NO, GSH assay, catalase, cytochrome c, comet assay, and caspase-3 were used to determine the effect of neutron radiation and also neutron and curcumin combination on the viability of cancer cells.

**Results:**

The results of cytotoxicity test showed that neutron irradiation with or without curcumin at 5, 10, 15, and 20 h reduced the survival of tumor cells. Moreover, the rate of apoptosis due to the neutron effect at different irradiation times enhanced with the increasing time.

**Conclusion:**

Due to the significant anticancer effect of curcumin in 3D culture, using this molecule before or after neutron therapy is recommended.

## INTRODUCTION

With increasing the use of radiation in medicine, industry, and research over the past few decades, the need for more radiation protection is essential^[^^1^^]^. Studies have shown the possible development of late-stage radiation complications, such as cancer, genetic damage, and leukemia, in radiation staff. This issue necessitates focusing on advanced research in the field of radiation protection. Radiotherapy is used by many medical centers for treating cancers, including breast cancer, the second most prevalent malignant tumor in women globally^[^^2^^,^^3^^]^. Data published by the Iranian Cancer Research Center demonstrated that one in 10-15 women is affected with this cancer^[^^4^^]^. 

Today, discovery of beneficial therapies and effective drugs with the least side effects in patients is on the agenda of many research centers around the world. Neutron therapy is a proposed method for treating some types of cancer^[^^5^^]^. Evidence has indicated that neutrons are more efficient than photons in the elimination of hypoxic tumor cells, and the amount of damage caused by these particles is much more less dependent on the cell cycle^[^^5^^]^. Owing to the larger relative biological effect of neutrons than photons, neutron therapy is now considered a treatment choice for treating slow-growing tumors such as breast cancer. A strategy to decrease the destructive adverse effects of neutrons is the reduction of the dose received using combination therapies such as biotherapy and neutron therapy. The main advantage of this method is the mitigation of systemic toxicity caused by radiation therapy due to the decrease of neutron dose received by patients^[^^6^^]^. 

One of the biomolecules with anticancer properties suggested for cancer treatment is curcumin, the main active ingredient in turmeric, which has extensive biological features such as anti-inflammatory, antioxidant, antidiabetic, and anticancer activity^[^^7^^,^^8^^]^. The 3D cell culture is a promising laboratory method used to assess the growth and viability of cancer cells exposed to chemicals, biomolecules, and radiation by creating a micro-environment closer to those in living organisms^[^^9^^]^. This study, for the first time, investigated the combined effect of neutron radiation produced by ^241^Am-Be neutron source and curcumin on breast cancer cells (MCF-7) using 3D cell culture.

## MATERIALS AND METHODS

**Materials and equipment**

MTT (Sisco Research Laboratories Pvt. Ltd., India), DMSO (Scharlau, Spain), antibiotic-antimycotic (Invitrogen, USA), sodium hydroxide hydrochloric acid, ethidium bromide, agarose, acetic acid, phosphoric acid, glycerol, NR dye, and Coomassie brilliant blue G-250 were purchased from Merck, Germany. Penicillin, phenol red, Triton X-100, Trypsin-EDTA, and Trypan blue were procured from Sigma-Aldrich, USA. DMEMF_12_, DMEM, and fetal bovine serum were obtained from Gibco, USA, and curcumin from Karen Pharmaceutical Company, Iran. Also, 24- and 96-well flat-bottom plates were purchased from Orange, Korea. Breast cancer cells (MCF-7, IBRC C10082) were acquired from the Cell Bank of Imam Hossein University, Tehran, Iran.

**Effect of neutron and curcumin on MCF-7 cytotoxicity using 3D culture**

***Encapsulation of cells in alginate***

To achieve a 3D environment, we encapsulated the MCF-7 cells in alginate particles. For this purpose, the alginate solution was prepared by dissolving 0.12 g of sodium alginate powder (Sigma-Aldrich, USA) in 10 mL of sodium chloride solution (0.9%). After filtering using a 0.22-μm plastic syringe filter, the alginate solution was added to a 96-well plate containing 2 × 10^4^ MCF-7 cells. Then the volume of the solution was reached 1 mL. Individualization and suspension of cells in alginate solution were performed using a 22-gauge plastic syringe. Alginate capsules were produced by injecting a mixture of cell-alginate to a bath of 100 mM of calcium chloride using a 22-gauge plastic syringe. Drops were released into the bath from a distance of 5 cm above the surface of bath. The capsules were allowed to be polymerized for 10 min. In the next step, following removal of calcium chloride, the capsules were washed three times with PBS solution (pH 7.4). The washing solution was replaced with 1 mL of DMEM containing 10% FBS. The cell capsules were incubated at 37 °C in 95% humidity and 5% CO_2_^[^^10^^]^.

 ***Depolymerization of alginate capsules and release of the cells***

Curcumin with the purity of 95% was procured from Karen Pharmaceutical Company. To achieve the final concentration of curcumin (i.e. 80 μM), 0.02 mg of curcumin was dissolved in sterile DMEM containing 1% antibiotic-antimycotic and incubated in a 5% CO_2_ incubator at 37 °C overnight to remove the biological contaminants. For performing experiments (GSH, catalase, and comet assay) in 3D culture, cells were exposed to 80-μM concentration of curcumin for 24 h, and then cell-containing capsules were depolymerized. Next, the capsules were carefully transferred to 2-mL microtubes, which washed thoroughly several times with PBS. Subsequently, 1 mL of 50 mM of sodium citrate solution was added to each microtube, and the tubes were incubated at room temperature for 20 min. For complete dilution of the capsules and release of the cells, the microtubes were centrifuged at 4 °C for 5 min. The supernatant was discarded, and the cell pellet was suspended in PBS. The microtubes were again centrifuged at 4 °C for 3 min. The supernatant was discarded, and the sediment containing the cells was used in the above-mentioned experiments^[^^9^^]^.

***Cell cultures and tests***

Based on the results of our previous study, the IC_50_ of curcumin was 80 μM^[11]^. This concentration was used to evaluate the combined effect of neutron radiation and curcumin on the cancer cell growth. The length of the cell culture vial/flask employed in the experiments was about 11 cm. Regarding the length of the BF3 detector (31 cm), its middle (15.5 cm) was considered as the sensitive point. The distance from the collimator entrance was determined by the formula (1-1). 

Collimator length (43 cm) – (detector middle [15.5] + vial length[11]/2) = 22 cm (1-1)

Cells encapsulated in alginate were incubated in flasks containing a culture medium with and without curcumin at a distance of 22 cm from the ^241^Am-Be source for 5, 10, 15, and 20 h. At these time periods, the encapsulated cells were exposed to neutrons at 3, 6, 9, and 12 mGy/h, respectively. Thereafter, the flasks containing the cells were transferred to an incubator. The total duration of cell exposure to neutrons and curcumin along with its storage in the incubator was 24 h. Effect of neutrons on the cytotoxicity of breast cancer cells in the presence and absence of curcumin under 3D culture and in the above conditions was performed using MTT, NR uptake, NO, GSH, catalase, cytochrome c, comet, and caspase-3 assays.

***Determination of cell viability by MTT assay***

MTT assay was performed as explained before^[^^12^^]^. Alginate capsules containing ~1 × 10^4^ cells were incubated in a flask (containing culture medium with 80 μM curcumin) while exposing to neutron radiation at a distance of 22 cm from the ^241^Am-Be source for 5, 10, 15, and 20 h. A similar flask without curcumin was used as a control. After the above times, the flasks containing cells were transferred to the incubator in 5% CO_2_ and 80% humidity at 37 °C. Total duration of cell contact with neutrons and curcumin in the incubator was 24 h. Thereafter, alginate capsules were transferred to a 96-well plate (three replicates), and then 20 μL of MTT solution (5 mg/mL) was added to each well. The plate was transferred to a CO_2_ incubator till dark blue crystals (formazan) were formed. After removing the content of each well and washing the wells with PBS, 200 μL of DMSO was added to each well. For complete dilution of precipitate, the plate was incubated in darkness at room temperature for 2–4 h. In this test, a culture medium was used as a negative control, and a culture medium containing cells was used as positive control. The light absorption of the wells was measured at 570 nm by a microplate reader (Biotek, USA). This experiment was repeated three times, and three wells (three times) were considered for each concentration. Cell viability was calculated by the following formula^[^^13^^]^: Vitality percentage of cells = 100× (a/b); where a and b indicate respectively the OD of the test sample and control minus the blank's OD.

***NR uptake assay***

NR uptake assay was used to verify the MTT assay results^[^^14^^]^. The NR test is based on the ability of live cells to absorb and accumulate NR dye in lysosomes^[^^12^^]^. The steps of incubation and radiation in NR uptake assay was similar to MTT assay. However, in this test instead of adding the MTT solution to the cell capsules transferred to the microplate, 4 μL of NR dye (5 mg/mL) was added to each well and incubated in the dark (5% CO_2_, 80% humidity, at 37 °C) for 1 h until the formation of red crystals. The solution in each well was then discarded and washed twice with PBS. Afterwards, 200 μL of fixing buffer (formaldehyde 37% [v/v] and CaCl_2_ 10% [w/v] in water) was added to each well and incubated similar to incubation conditions mentioned in MTT test. After 1 min, 100 μL of solubilizing buffer (acetic acid 0.5%) was added, and the plate was kept on a shaker in the dark for 20 min. The absorbance was measured at 540 nm by a microplate reader (Biotek). The inhibition percentage of cell growth was calculated using the following formula: percentage of cell cytotoxicity = 1-(100× [a/b]); where a and b denote respectively the OD of the test sample and the control minus the blank's OD.

***NO assay***

No determination was performed as described before^[^^15^^,^^16^^]^. The steps of NO assay were similar to the MTT assay. However, unlike MTT assay, in this test, 100 μL of the media was transferred to a 96-well plate and mixed with an equal volume of Griess reagent (0.04 g/mL in PBS, pH 7.4; Sigma-Aldrich). The mixture was maintained at room temperature for 10 min. Absorbance was measured at 520 to 550 nm by a microplate reader (Biotek). In the treated cells, NO concentration (μM/mL) was calculated using sodium nitrite standard curve.

***GSH assay***

GSH test was performed as explained previously^[^^17^^]^. The incubation and radiation steps of this test was conducted similar to NO assay. In this assay, unlike NO test, encapsulated cells containing 5 × 10^5^ cells were transferred to 1.5-mL tubes for testing. The lysis buffer (200 μL) was added to each well, and protein concentration was evaluated by Bradford assay^[^^17^^]^. Upon transferring the 40 μL of the obtained solution to new tubes, 40 μL of trichloroacetic acid solution (10%) was added to each tube, and the plate was stored at 4 °C for 2 h. The centrifugation was performed at a speed of 1500 rpm for 15 min, and the supernatant was transferred to a clean tube. Finally, 75 μL of lysis buffer, 55 μL of Tris-HCl buffer (pH 8.5), and 25 μL of dinitrothiocyanobenzene were added to 20 μL of the supernatant. OD of the samples was measured at 412 nm.

***Catalase enzyme activity assay***

This assay was conducted according to a formerly described method^[^^18^^]^. The steps of catalase test were similar to GSH test, except that after protein measurement by the Bradford method, the content of the flasks were mixed with 50 μL of the lysis buffer (20 μL of deuterium-depleted water and 25 μL of 15% H_2_O_2_). Then the flasks containing 100 μL of potassium dichromate solution was incubated at 37 °C for 2 min. After a pink color was observed on the surface of the solution, samples were incubated at 100 °C for 10-15 min until a green color appeared. The tubes were spun, and 150 μL of the solution in each well was transferred to a 96-well plate. The OD was measured at 570 nm using a plate reader (Biotek).

***Cytochrome c assay***

The initial steps of the cytochrome c test were performed the same as GSH test. In this assay, capsules containing ~1 × 10^6 ^cells were transferred to 1.5 mL tubes for testing. The cells were dissolved in 1 mL of the purification buffer (Tris-HCl), incubated at 0 °C on ice for 10 min and homogenized in a Dounce tissue grinder. The homogenized samples were transferred to a new 1.5-mL tube and centrifuged at 700 ×g at 4 ° C for 10 min. The supernatant was transferred to new 1.5-mL tubes and centrifuged at 10,000 ×g at 4 ° C for 30 min. Protein concentration was measured using Bradford, and OD was measured at 495 nm.

***Alkaline comet*** ***assay***

This method was performed according to a procedure performed before^[^^19^^]^. The steps of the alkaline comet test were similar to the MTT method, except that after receiving the radiation and incubating in an incubator, the capsules containing 12 × 10^4^ cells were transferred three times to 1.5-mL tubes. Then 200 μL of PBS was added to each tube containing the sediment of the cells released from the dissolved alginate capsules, and the cells were isolated using a needle. Slides were covered by normal melting agarose (1% [v/v]) and incubated at 4 °C for 10 min. Cell suspensions were mixed with low melting agarose (1% [v/v]; 1:2 ratios) and poured onto the slides. To create a layer of cells on each slide, a coverslip was placed on the slide and stored in a refrigerator for 10 minutes. After taking the slides out of the refrigerator, their coverslips were gently removed. To do cell lysis and nucleus distraction, all slides were incubated in a fresh and cold lysis buffer (NaCl, 2.5 M; EDTA, 100 mM; Tris, 10 mM; NaOH, 0.2 M; Triton X-100, %1; pH 10) at 4 °C for 16-18 h. The slides were then washed twice with electrophoresis buffer (89 mM of Tris base, 0.4 M of boric acid, and 8 mM of EDTA) at 4 °C for 20 min and electrophoresed at 4 °C (25 V and 300 mA) for 45 min. For neutralization, the slides were incubated in a neutralizing buffer (0.04 M of Tris, pH 7.5) for 10 min. After incubating with 100 μL of ethidium bromide (20 μg/ mL) at room temperature for 10 min, the slides were rinsed two times with water and observed by an inverted fluorescent microscope (Nikon, Japan). The results were eventually analyzed statistically.

***Caspase-3 activity test***

Caspase-3 activity was evaluated using the commercial Caspase-3/CPP32 laboratory kit (sigma-Aldrich, USA) according to the manufacturer's instructions^[^^10^^]^. The steps for evaluating the activity of caspase-3 were the same as the cytochrome c test. However, in this test, after dissolving the alginate capsules, the resultant cell precipitate and cell plate were suspended in 100 mL of a cold lubricating buffer (10 mM of Tris, 1 mM of EDTA, and 1% Triton X-100; pH 7.4) and incubated on ice for 20 min. In the next step, 5 μL of DEVD-pNA (200 µM) was added to each sample and incubated at 37 °C for 1 h. Finally, the absorbance of the samples was measured at 405 nm.

**Statistical analysis**

Comparison of the anticancer effect of neutron in the presence or absence of curcumin on MCF-7 breast cancer cells was evaluated by GraphPad InStat (version 6) software and two-way ANOVA. Differences were considered to be statistically significant at *p *< 0.05. All experiments were conducted three times.

## RESULTS

 **MTT assay**

**Fig. 1:**
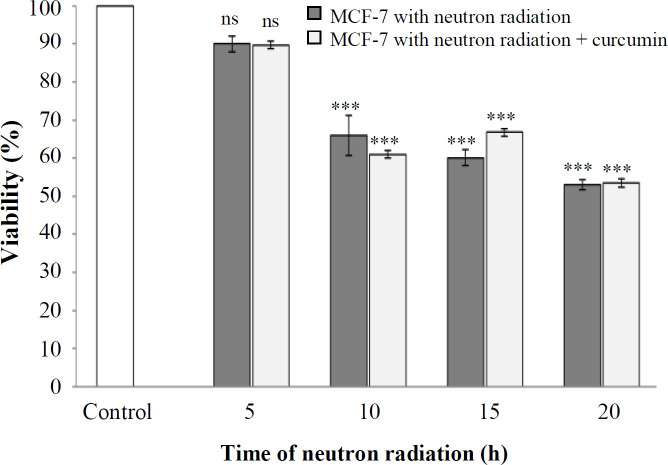
The percentage of cell viability at different times of neutron irradiation in the presence and absence of curcumin using the MTT assay (^***^p < 0.001 compared with control). ns, not significant

The results of MTT assay showed that the survival rate of MCF-7 cells following neutron radiation at 5, 10, 15, and 20 h was 90%, 65.96%, 62.1%, and 53.06%, respectively. This rate was 89.7%, 61.3%, 64.9%, and 50.2% in the presence of curcumin (80 μM) at the mentioned times (Fig. 1). Also, neutron radiation had a remarkable effect on the viability of cells in the presence and absence of curcumin as compared to the control, except for 5 h (Fig. 1). Analysis of the results also indicated that the effect of neutron radiation on MCF-7 cell viability in the presence and absence of curcumin at times 5, 10, 15, and 20 h was insignificant (*p *≤ 0.05)

**NR uptake assay**

**Fig. 2:**
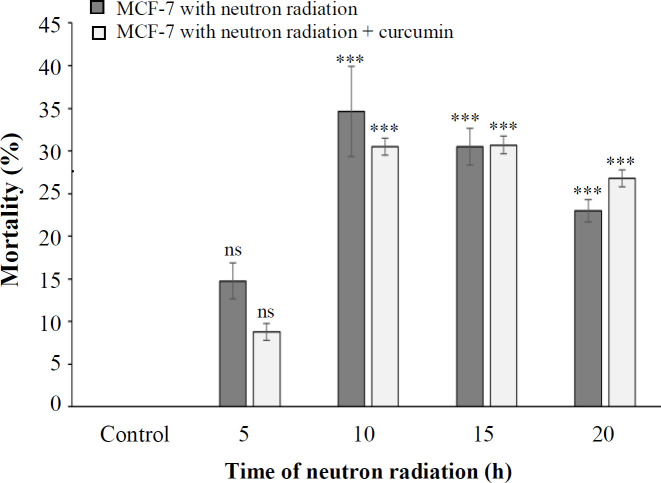
The percentage of cell mortality at different times of neutron irradiation in the presence and absence of curcumin using a NR test (^***^p < 0.001 compared with control). ns, not significant

Based on the NR uptake assay, the growth inhibition percentage of MCF-7 cells on neutron radiation at 5, 10, 15, and 20 h was 14.76%, 36.63%, 30.53%, and 22.96%, respectively. Following neutron irradiation in the presence of curcumin, this rate was 8.76%, 30.5%, 30.7%, and 26.8%. The results also demonstrated that the neutron radiation in the presence and absence of curcumin at all times had a significant inhibitory effect on the growth of cells except for 5 h (Fig. 2). However, the results of neutron radiation at the above-mentioned times in the presence and absence of curcumin were not significantly different.

**NO assay**

**Fig. 3:**
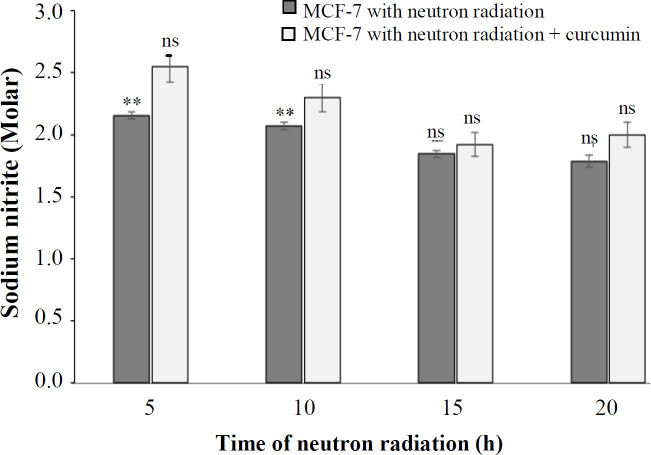
The percentage of NO generated at different times of neutron irradiation in the presence and absence of curcumin using the NO assay (^**^p < 0. 01 compared with control). ns, not significant

The amount of NO releasing from MCF-7 cells as a result of neutron irradiation at 5, 10, 15, and 20 h was 2.15, 2.06, 1.83, and 1.78 (molarities of sodium nitrite), respectively. As depicted in Figures 1-3, the amount of NO released from cells by neutron irradiation at all times was nonsignificant (*p *> 0.05). The amount of NO released from the cells under neutron irradiation in the presence of curcumin was 2.55, 2.31, 1.29, and 2.02. This effect was significantly 

different from the control at 5 and 10 h. These results showed that the effect of neutron radiation on MCF-7 cell growth with curcumin compared to without curcumin at the above-mentioned times was not significantly different.

**Catalase enzyme activity assay**

**Fig. 4:**
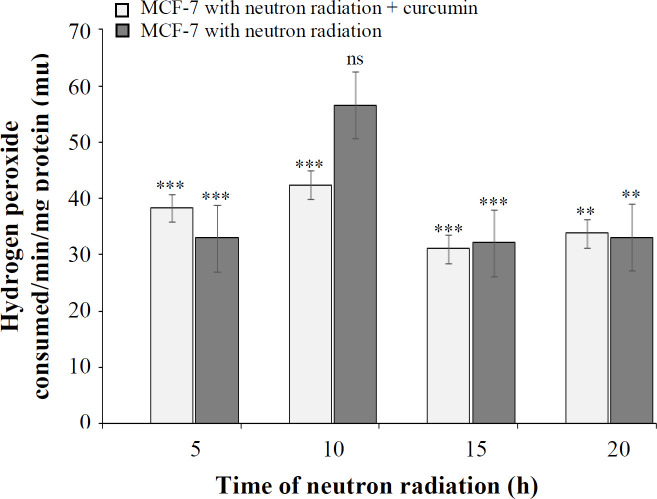
The percentage of catalase at different times of neutron irradiation in the presence and absence of curcumin using the catalase test (^**^p < 0.01, ^***^p < 0.001 compared with control). ns, not significant

Based on the result of this method, the amount of cellular catalase generated by neutron irradiation at 5, 10, 15, and 20 h was 32.94, 57.43, 32.12, and 33.09 μmoles of hydrogen proxide consumed/min/mg protein, respectively. As shown in Figures 1-4, at all times, except for 10 h, catalase level in the treated cells was considerably higher than that of the untreated cells. The catalase activity after neutron irradiation in the presence of 80 μM of curcumin at 5, 10, 15, and 20 h was 38.32, 42.42, 31.03, and 33.77 μmoles of hydrogen proxide consumed/min/mg protein, respectively. The produced catalase was not significantly different from the control at all times. The result also showed that the effect of neutron radiation in the presence of curcumin compared to the absence of curcumin was not significantly different, except for 20 h.

**GSH assay**

**Fig. 5:**
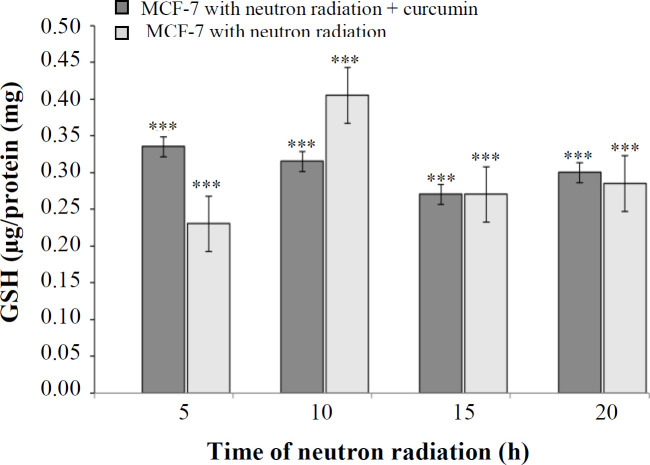
The percentage of GSH at different times of neutron irradiation in the presence and absence of curcumin using the GSH assay (^***^p < 0.001 compared with control)

The results of GSH assay revealed that the cellular GSH level produced under neutron radiation at 5, 10, 15, and 20 h was 0.23, 0.40, 0.27, and 0.28 (μg of GSH/mg protein), respectively. The amount of GSH produced after neutron radiation in the presence of curcumin at the above irradiation times was 0.33, 0.31, 0.27, and 0.30 (μg of GSH/mg protein), respectively. As shown in Figures 1-5, neutron radiation in the presence and absence of curcumin significantly increased the level of cellular GSH at all times. The result also showed that the effect of neutron radiation on MCF-7 cell growth in the presence and absence of curcumin at all times, except for 5 h, was nonsignificant.

***Cytochrome c assay***

**Fig. 6:**
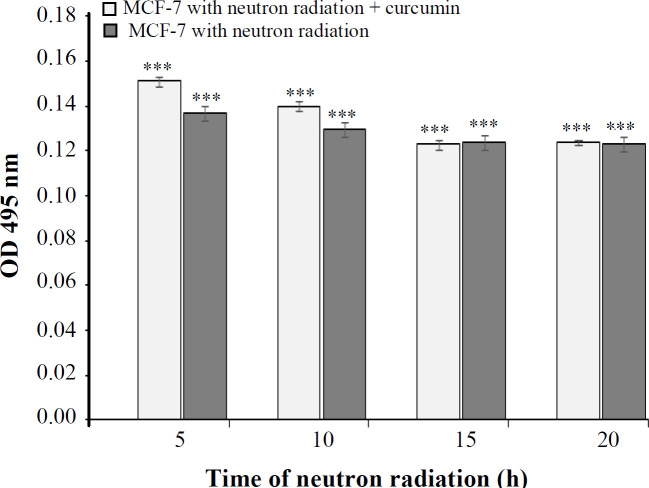
The percentage of cytochrome c released from mitochondria into cytosol at different times of neutron irradiation in the presence and absence of curcumin using the cytochrome c assay (^***^p < 0.001 compared with control)

This assay exhibited that following neutron irradiation at 5, 10, 15, and 20 h, the amount of cytochrome c released into the cytosol in MCF-7 cells under 3D culture was 0.137%, 0.130%, 0.124%, and 0.123%, respectively. However, after the neutron irradiation, this amount was 0.151%, 0.40%, 0.123%, and 0.124% in the presence of 80 μM of curcumin. As shown in Figures 1-6, the presence of curcumin had a significant inhibitory effect on the cell growth following neutron radiation at all times. Moreover, the results showed that the presence of curcumin in combination with the neutron radiation had no significant effect on the cell growth inhibition.

**Alkaline comet assay**

**Fig. 7:**
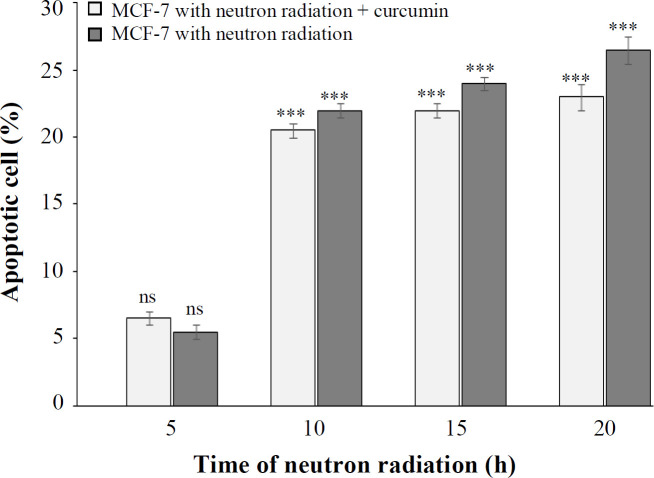
The percentage of apoptosis at different times of neutron irradiation in the presence and absence of curcumin using the comet assay (^***^p < 0.001 compared with control). Ns, not significant

The alkaline comet assay was used to investigate the mechanism of cell death induced by neutrons on MCF-7 cells. The induction rate of apoptosis at neutron irradiation times of 5, 10, 15, and 20 h on MCF-7 cells in 3D culture was 5.5%, 22%, 24%, and 26.5%, respectively. This rate was 6.5%, 20.5%, 22%, and 23% in the presence of curcumin at the mentioned irradiation times (Figs. 1-7). Statistical analysis showed that the effect of neutron radiation on the cell death induction in the presence and absence of curcumin was significant at all times, except for 5 h (*p * 0.05). Furthermore, based on the results, the effect of neutron radiation on the cell death induction did not depend on the presence or absence of the curcumin. 

**Caspase-3 assay**

**Fig. 8:**
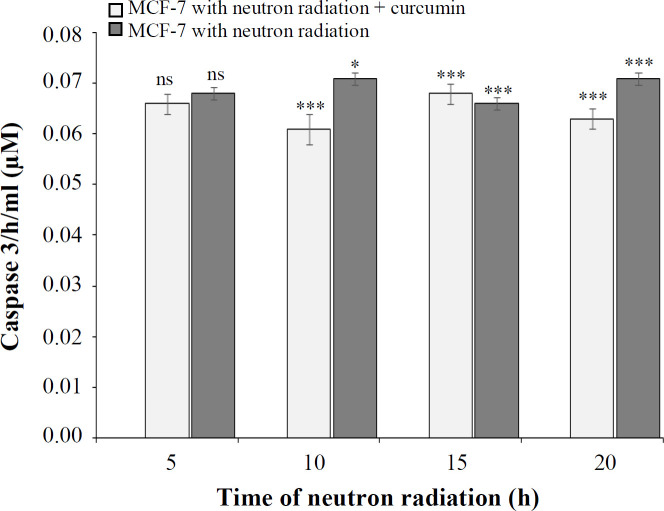
The percentage of caspase-3 activity at different times of neutron irradiation in the presence and absence of curcumin using caspase-3 assay (^*^p < 0.05, ^***^p < 0.001 compared with control). ns, not significant

The results of this assay showed that the effect of neutron radiation on the activity of caspase-3 in MCF-7 cells at irradiation times of 5, 10, 15, and 20 h was 0.068, 0.071, 0.066, and 0.071 μM, respectively. This effect of neutrons in the presence of curcumin at the times mentioned was 0.066, 0.061, 0.068, and 0.063 μM, respectively. Results also demonstrated that the production of this enzyme in the cells exposed to neutron irradiation at different times, except for 5 h, was significant as compared to the control (*p * 0.05). (Figs. 1-8). However, the effect of neutron irradiation in the presence and absence of curcumin on MCF-7 cells at the above-mentioned times was insignificant.

## DISCUSSION

Cancer is a major medical problem threatening human health worldwide. Breast cancer is the most common cancer prevalent among women^[^^2^^,^^3^^,^^10^^]^. Evidence indicates that treatments such as surgery, chemotherapy, and radiotherapy play a limited but important role in treating this disease. To increase the efficiency of treatment and duration of patients’ life, new cancer treatment strategies such as combined methods have been taken into account^[^^6^^]^. One of the advantages of using these novel methods is decreased systemic toxicity from chemotherapy, radiation, or radiotherapy due to the reduction of the dose received by the patient^[^^6^^]^.

Curcumin in turmeric is a candidate molecule proposed alone or in combination for treatment of cancer. Results of studies have shown that this molecule has broad biological (anti-inflammatory, antioxidant, antidiabetic, and anticancer) properties^[^^8^^,^^7^^]^. In this study, effect of neutron radiation alone and the synergistic effect of neutron radiation and curcumin on MCF-7 breast cancer cells growth in a 3D culture medium were studied. MTT, NR uptake, NO, GSH, catalase, cytochrome c, comet, and caspase-3 assay were used to determine the effect of neutron radiation and also neutron and curcumin combination on the cytotoxicity of cancer cells. Using the MTT, cytotoxicity caused by the neutron radiation as well as neutron radiation combined with curcumin (80 μM) were evaluated, and the results were confirmed by the NR. 

The results of MTT showed that neutron irradiation at 5, 10, 15, and 20 h reduced the viability of tumor cells by 90%, 65.96%, 62.1%, and 53.06%, respectively. However, the cell viability under neutron radiation in combination with curcumin at these times was statistically insignificant as compared to neutron radiation alone, which was 89.7%, 61.35%, 64.9%, and 50.2%, respectively. The comet assay results showed that the rate of apoptosis induction due to neutron effect at irradiation times of 5, 10, 15, and 20 h respectively enhanced with the increasing the irradiation time. Also, statistical analysis indicated that the rate of apoptosis due to the combined effect of neutrons and curcumin was insignificant as compared to the effect of neutrons alone. In 2011, Veeraraghavan *et al.*^[^^20^^]^ evaluated the effect of combined therapy with curcumin and radiation on pancreatic cancer cells. They observed minimum viability, maximum cytotoxicity, and strong apoptosis induction after combining 100 nM of curcumin with radiation. Moreover, their findings exhibited that the addition of curcumin before irradiation effectively inhibited the G2/M phase of the cell cycle^[^^20^^]^. Zhan et al.^[^^21^^]^ have also reflected that curcumin increases the sensitivity of MCF-7 cells to chemotherapy drugs and concluded that curcumin can be an intermediate molecule and a suitable candidate for using in the combinational cancer therapies^[^^22^^]^. Damondaran *et al.*^[^^23^^]^ studied the effect of 5 μM of curcumin on p53 mutant prostate cancer cells and compared the results with those of the combined treatment of curcumin (2 μM) with x-ray radiation. Their observations showed that curcumin is a potent radiation sensitizer that inhibits the growth of prostate cancer cells and reduces cell survival factors as a result of radiation and increases the sensitivity of radiation in tumor cells. They also denoted that radiation therapy in combination with curcumin, compared to radiation alone, could inhibit tumor growth and increase cell death in various types of cancer^[^^23^^]^. 

In this study, we used the ^241^Am-Be source with 5-Curie activity, which has been exhibited to be fast and one of the most widely used neutron sources^[^^24^^]^. Neutrons cause various interactions depending on their energy level when exposed to a living cell. Fast neutrons in contact with living tissue cells produce particles such as protons, alpha, and photons that have a destructive effect on DNA^[^^25^^]^. This neutron damages the cell membrane and activates cell death pathways^[^^24^^]^. Investigations have signified that neutrons are involved in hypoxia-induced cell death. This feature has led to the selection of neutrons for the treatment of low-growth tumors such as prostate cancer^[^^25^^]^. In general, neutrons are much more effective in destroying tumor cells than radiation with low linear energy transfer, such as gamma, X, electron, and so forth^[^^26^^]^. In our previous study, we evaluated the effect of curcumin on the MCF-7 cell growth in 3D culture in alginate particles and displayed that 80 μM concentration of curcumin could reduce the viability of breast cancer cells by inducing necrosis and mainly apoptosis. The results of our former study also showed that curcumin decreases the production of nitrite oxide in cells and increases the production of catalase and glutathione^[^^11^^]^. In an earlier study, Souto *et al.*^[^^27^^]^ investigated the effects of fast neutrons produced by the ^241^Am-Be source on polycarbonate materials and disclosed that these neutrons at a dose rate of 0.5-20 millisievert could destroy carbon and oxygen bonds in materials. Saeed *et al.*^[^^28^^]^ assessed the effect of very low doses of fast neutrons (0.009 Gy) on ​​the output of ^241^Am-Be source with 5 Curie activity *in vivo* on erythrocyte lipid membranes and proteins. Their results exhibited that the greatest effect of neutrons on cell membranes is due to the impact of these particles on methyl and methylene groups. Nafee *et al.*^[^^29^^]^ reported that neutrons alter or inactivate the protein responsible for cell transcription by breaking the C–O bond in RNA. Examining the molecular formula of curcumin (C_21_H_20_O_6_) shows that this molecule has a hydrocarbon structure and multiple bonds, including C–O and C–H^[^^30^^]^. According to the reports of Souto *et al.*^[^^27^^]^, Saeed *et al.*^[^^28^^]^, and Nafee *et al.*^[^^29^^]^, neutrons produced by the ^241^Am-Be source could destroy and damage C–O and C–H bonds. The induction of apoptosis in neutron-exposured cancer cells in this study is consistent with Nafee et al.’s study^[^^29^^]^.

The results of evaluating the effect of neutrons and the combination of neutrons and curcumin on the production of NO, catalase, and GSH under the influence of different irradiation times demonstrated no statistically significant difference in MCF-7 cell. Moreover, curcumin reduced NO level and increased the production of catalase and GSH. The results of neutron effect alone and the combined effect of neutron and curcumin on the release of cytochrome c from the mitochondria and also the activation of caspase-3 showed that the amount of cytochrome c release in the cytoplasm had an upward trend, depending on the irradiation time and caspase-3 activation. The results of these two experiments were statistically significant compared to the control but insignificant compared to each other. Our results were in line with those of Damondaran *et al.*^[^^23^^]^ who showed that the combined therapy with curcumin and radiation causes the activation of caspase-3 in cells. 

In conclusion, the findings of this study reveal that the neutron source ^241^Am-Be with the applied dose can destroy the C–O and C–H bonds of curcumin, resulting in induction of cell death in breast cancer cells, mainly via apoptosis. Based on the results of MTT test and its comparison with other tests (i.e. cytochrome c test, alkaline comet assay, and caspase 3 activity test), which displays the cell death apoptosis, the rate of death due to necrosis was much higher than apoptosis; however, neutron induces apoptosis in breast cancer cells. Owing to the significant anticancer effect of curcumin in 3D culture, the use of this molecule before or after neutron therapy is recommended.

## DECLARATIONS

### Acknowledgments

The authors gratefully acknowledge the members of the Imam Hossain University for their insights and assistance.

### Ethical statement

Not applicable.

### Data availability

The analyzed data sets generated during the study are available from the corresponding author on reasonable request.

### Author contributions

SZ: study concept and design, acquisition of data, analysis and interpretation of data, drafting of the manuscript, statistical analysis, administrative, technical, and material support; MSB: study concept and design, acquisition of data, critical revision of the manuscript for important intellectual content, and statistical analysis; JZ: study concept and design and statistical analysis; MS, analysis and interpretation of data; AHNM, critical revision of the manuscript for important intellectual content, administrative, technical, and material support; MM, critical revision of the manuscript for important intellectual content, administrative, technical, and material support; HKA, critical revision of the manuscript for important intellectual content, administrative, technical, and material support.

### Conflict of interest

The authors declare that they have no conflicts interest.

### Funding/support

This research was conducted at Islamic Azad University (Central Tehran Branch), but all costs were provided privately.
